# Genetic Diversity and Differentiation of Silkworm (*Bombyx mori*) Local Germplasm Resources in China and Uzbekistan

**DOI:** 10.3390/insects15121020

**Published:** 2024-12-23

**Authors:** Xia Xu, Xin Du, Jine Chen, Lusong Yao, Xiuling He, Linbao Zhu, Shaofang Yu, Valiev Sayfiddin Tojiddinovich, Baxtiyar Ubaydullayevich Nasirillayev, Ismatullaeva Diloram Adilovna, Khudjamatov Safarali Khasanboy ugl, Yongqiang Wang

**Affiliations:** 1 Institute of Sericulture and Tea, Zhejiang Academy of Agricultural Sciences, Hangzhou 310021, China; xuxia@zaas.ac.cn (X.X.); duxin@zaas.ac.cn (X.D.); chenje@zaas.ac.cn (J.C.); yaols@zaas.ac.cn (L.Y.); hexl@zaas.ac.cn (X.H.); zhulb@zaas.ac.cn (L.Z.); yusf@zaas.ac.cn (S.Y.); 2 Scientific Research Institute of Sericulture, Tashkent 100169, Uzbekistan; stvaliev@mail.ru (V.S.T.); bahtiyor6503@gmail.com (B.U.N.); toir.begmatov0913@gmail.com (I.D.A.); alixudjamatov92@gmail.com (K.S.K.u.)

**Keywords:** *Bombyx mori*, germplasm resources, production traits, genetic diversity, sericulture, genomic resequencing

## Abstract

Silkworm (*Bombyx mori*) is an economically significant insect that produces silk and makes important contributions to the development of silk-producing countries. The genetic diversity and unique adaptive traits of silkworm germplasm resources form the foundation for breeding efforts. In various geographical regions, silkworm have developed distinct traits through long-term adaptive selection. In this study, we focused on identifying and evaluating representative silkworm germplasm resources from both China and Uzbekistan. We discovered notable differences in the morphology and production traits of local silkworm strains, likely influenced by differing geographical environments. It is also possible that China has a long history of silkworm rearing and after a long time of breeding selection, the diversity of silkworm morphology is less than that of Uzbekistan, but the production characteristics are more suitable for silk utilization. Phylogenetic analysis based on genomic comparisons revealed that Uzbekistan’s local silkworm strains are positioned between China’s local and improved strains. This suggests that Uzbekistan silkworms primarily originated from China. The further analysis of genetic diversity and strain differentiation highlights the unique differences of each country’s silkworms, especially in terms of genetic diversity on chromosome 1 (sex chromosome). By identifying and evaluating these germplasm resources and linking them to unique advantageous traits, it provides a scientific basis for improving cocoon silk quality and optimizing sericulture productivity.

## Simple Summary

Germplasm resources serve as the foundation for breeding, with innovations fueling the development of new varieties. Notable differences exist in the morphology and production traits of representative local silkworm strains from China and Uzbekistan. The phylogenetic analysis of the genomic comparisons indicated that the local silkworm strains in Uzbekistan occupy a position between the local and improved strains from China. By identifying and evaluating these silkworm germplasm resources and selecting unique advantageous traits, we establish a theoretical framework to significantly enhance the quality of cocoon silk and improve the overall efficiency of sericulture.

## 1. Introduction

Sericulture boasts a history spanning 5000 years, during which various germplasm resources of the silkworm, *Bombyx mori*, have emerged through dissemination and development. Germplasm resources, also referred to as genetic resources, denote the genetic material passed from an organism’s parents to its offspring, which is often represented by specific strains, such as ancient local strains and newly bred strains [1]. As the primary source of the world’s silk, China has also facilitated the spread of the silkworm (*Bombyx mori*) to Western countries through the Silk Road [2]. Uzbekistan plays a vital role as a key country along the Silk Road, dominating the Central Asian silk industry. Over the long term, sericulture practices in various regions have evolved due to geographical isolation. Through natural selection under diverse environmental conditions and artificial selection with different targets, trait variation has accumulated. Consequently, rich silkworm germplasm resources with genetic diversity have gradually developed [3]. Both China and Uzbekistan are the main silk producing countries. Uzbekistan is situated at approximately 41° north latitude, while China’s major sericulture-producing regions lie below 35° north latitude. As a result, the two countries possess distinct germplasm resources and techniques due to significant differences in their silk production environments and climates [4].

Germplasm resources form the foundation of breeding, with innovations propelling the development of new strains [3]. For example, local strains have evolved and selected naturally in a particular area. They often have the ability to adapt to specific environments and can perform well in local environments. Genetic stocks are a library of biological materials containing specific genetic information. These repositories typically include a variety of genetic resources for scientific research, breeding, and biodiversity conservation. Improved strains are bred through genetic improvement techniques (e.g., hybridization, selective breeding, etc.). These strains usually have higher yields, better disease resistance and adaptability, and can meet the needs of agricultural production. Wild silkworms grow in the natural environment, without artificial selection and improvement. Wild silkworms typically have high genetic diversity and adaptability, and are an important resource for breeding and biological research. As society advances and technology progresses, the innovation of silkworm germplasm resource has evolved from local resource mining and the cultivation of naturally obtained strains to breeding by genetic crosses between strains, hybridization, recombination, induced mutation, and modern genetic engineering [5,6,7,8]. Strategies such as systematic isolation and multi-generation selection have led to the development of high-yield silkworm strains, expediting the breeding of superior-quality specimens [9,10]. Techniques like X-ray-induced chromosome fragment translocation and sex-linked lethal balance systems have further enhanced the breeding of commercial silkworms [11].

To develop silkworm strains that perform well, a wide range of dietary germplasm resources have been screened from natural variants. This has provided a genetic basis for the successive breeding of edible silkworms on artificial feeds [12]. Moreover, advancements in molecular biology have enabled scientists to genetically modify silkworm germplasm resources, significantly contributing to innovation in sericulture [13,14,15]. For example, genetic mutant lines of naked pupae have been selected to meet the needs of silkworms as bioreactors [16,17]. The γ-ray-induced interlocking balanced lethal system has made it possible to only rear male silkworms [18]. Genome resequencing for germplasm assessment provides greater insight into genetic diversity and strain differentiation [19,20]. Researchers created a pan-genome map of silkworm, which is crucial for advancing functional genome research and germplasm innovation [3]. Coupled with modern molecular techniques, the targeted screening of genes that make up production traits could pave the way for breeding suited to diverse regions and requirements.

In this study, we evaluate the germplasm resources of representative local silkworm strains from China and Uzbekistan. In particular, we analyze genome data to identify potential target genes associated with dominant traits. We anticipate that the distinct history of silkworm breeding will be reflected in the phenotypic and genetic variations among the strains. Specifically, we hypothesize to find more variation in traits and genetic make-up of the Uzbekistan strains that have a more recent origin and undergone a less uniform selection regime. Our findings will provide valuable insights for silkworm breeding initiatives aimed at improving cocoon quality and enhancing overall sericulture productivity.

## 2. Materials and Methods

### 2.1. Silkworm Strains

Fifteen representative local silkworm strains from Uzbekistan were bred and provided by the Tashkent scientific research institute of sericulture, including: AGU, C13, C14, GE143, GUZAL, IPAKCHI3, L51, L6m, MARGILAN, MG, SANIISH8, SANIISH30, SZEM4, UZNIISH9, and YAQUB. Fifteen representative local silkworm strains from China were bred and provided by the Zhejiang Academy of Agricultural Sciences, including D12, D16, D18, D19, D21, D22, Linhai No.20, Sangcan (Hu), Taihu, Wulong, Yiwu, Yuhang No.2, Yuhang No.7, Yuhang 24, and Zhugui. A comparison was made with 1078 silkworm genomes from the National Center for Biotechnology Information (NCBI) open database, including 205 local strains, 194 improved varieties, 632 genetic stocks, and 47 wild silkworms [3].

### 2.2. Trait Investigations

Trait investigations were conducted on the 15 representative local silkworm strains from both Uzbekistan and China. The investigated traits included physical appearance (egg color, egg shape, cocoon shape, moth eye color, moth wing markings), production performance (incubation time, larval stage time, pupal stage time, hatching uniformity, molting and exuviating uniformity, cocoon weight, relaxation rate, cocoon filament length, cocoon filament size), and reproductive ability (egg attachment, egg number, normal egg, moth emergence uniformity). Thirty individuals were randomly selected for each strain and further investigated for each trait. Among them, 30 pairs of silkworm adults were randomly selected from each strain to mate and lay eggs. Then, the eggs were collected and mixed evenly, and 200 mg was weighed for further trait analysis. In terms of uniformity during silkworm production, more than 90% of the same behavior was noted as ‘uniform’, 80~90% was noted as ‘relatively uniform’, <80% was noted as ‘non-uniform’. Results are presented as means with error bars as ± SEM (standard error of the mean). A two-tailed Student’s *t*-test was used to analyze differences, with * *p* < 0.05, ** *p* < 0.01, *** *p* < 0.001, n.s. *p* > 0.05.

### 2.3. Genomic DNA Extraction and Genome Resequencing

Thirty pairs of silkworm adults were randomly selected from each strain to mate and lay eggs. Then, the eggs were collected and mixed evenly, and 200 mg was weighed for genomic DNA extraction. Eggs were ground into powder and added to a genome extraction buffer containing proteinase K (10 mM Tris-HCl, 100 mM EDTA, 1% SDS) and treated with RNaseA for DNA extraction using phenol: chloroform: isoamyl alcohol (25:24:1). Subsequently, the genome DNA was purified by isopropanol precipitation. Genome DNA was resequenced using the DNBseq sequencing platform of BGI (https://www.genomics.cn/). The coverage was about 95% for each sample. The raw sequencing data were filtered, including removing adaptor sequences, contamination, and low-quality reads, to obtain valid data. We first went through a series of data processing to remove contamination and obtain valid data by SOAPnuke (2.0) software. SOAPnuke software filter parameters: “-*n* 0.001 -| 10 -q 0.5 --adaMR 0.25 --polyX 50 --minReadLen 150” [21].

### 2.4. Phylogenetic Analysis

The genome resequencing data of Uzbekistan representative local silkworm strains (BioProject: PRJNA1196356) were compared with the Variant Call Format (VCF) data of 1078 silkworm genomes (CNGB: CNP0002456) to construct a phylogenetic tree [3]. The Soapnuke (2.0) software was used to filter the genome resequencing data, removing adapter contamination and low-quality reads. The filtered CleanData was aligned to the reference genome using the Burrows–Wheeler Aligner (BWA). Subsequently, the Genome Analysis Toolkit (GATK) was used to detect the single nucleotide polymorphisms (SNPs) and Indels for all samples, and each variant site was quality-controlled and filtered, followed by phylogenetic tree construction using the FastTree (2.1.11) software with Maximum Likelihood (ML). This maximized the probability of observation data on the tree. The FastTree basic command format was FastTree-nt sequences.fasta > tree.nwk [22,23].

### 2.5. Single Nucleotide Polymorphism Analysis

The *Pi* (π) analysis of strain nucleotide diversity involved calculating the SNP within sliding windows of different strains according to the formula:

$$Pi\left(\pi \right)=\sum_{ij}xixj\pi ij=2\times \sum_{i=1}^{n}\sum_{j=1}^{i-1}xixj\pi ij$$

where *xi* and *xj* represent the corresponding frequencies of the *i* and *j* sequences, respectively. *xij* is the percentage of different sites between the two sequences, and *n* is the total number of samples. A window size of 100,000 bp and a step size of 50,000 bp were set to calculate the *Pi* value for each strain. The *Pi* ratio was calculated by dividing the *Pi* value of the 15 Uzbekistan representative local silkworm strains by the 15 China representative local silkworm strains, considering regions outside the 95% confidence interval as significant difference regions. Regions above the 95% confidence interval were considered as selective regions for Uzbekistan, and regions below the 95% confidence interval were considered as selective regions for China. *Pi* analysis of strain nucleotide diversity can reveal differences in genetic diversity among strains. Strains subjected to artificial selection tend to have relatively lower genetic diversity with smaller *Pi* values, whereas wild strains typically display higher genetic diversity with larger *Pi* values.

### 2.6. Strain Differentiation Analysis

The fixation index of subdivision (*Fst*) is an index to measure the degree of strain differentiation given by the formula:

$$Fst=\frac{1}{n}*\sum \frac{{pi}^{2}-H^{2}}{1-H^{2}}$$

where *H* represents the frequency of a given allele, *pi* is the above single nucleotide polymorphism value, and *n* is the total number of samples. A window size of 100,000 bp and a step size of 50,000 bp were set to calculate the *Fst* value for regions shared by the two strains. Values above the 95.00% interval indicated significant differentiation between the strains, i.e., selective regions. The fixation coefficient *Fst* reflects the level of allelic heterozygosity in a strain which is influenced by different factors, such as mutation, genetic drift, inbreeding, and selection. The *Fst* value ranges from 0 to 1. An *Fst* value of 0 means that the two strains are not differentiated, and an *Fst* value of 1 indicates that the two strains are fully differentiated, i.e., do not share alleles at all. A further analysis was performed for regions above the 95% interval. Gene alignment annotation was performed with the known silkworm genome database (http://silkworm.genomics.org.cn) using Bowtie2 (2.5.2) software. The Gene Ontology (GO) terms of genes were identified using the *InterProScan* v5.41-82.0 program with default parameters.

## 3. Results

### 3.1. Morphology and Production Traits

**Figure 1:**
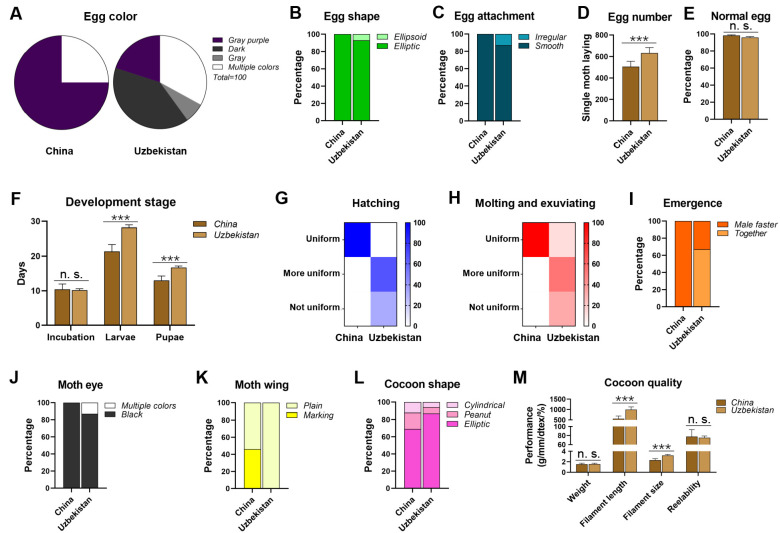
**Morphology and production traits of 15 representative strains from Uzbekistan and China.** (**A**) Egg color. (**B**) Egg shape. (**C**) Egg attachment. (**D**) Egg number. (**E**) Normal egg. (**F**) Development stage. (**G**) Hatching. (**H**) Molting and exuviating. (**I**) Emergence. (**J**) Moth eye. (**K**) Moth wing. (**L**) cocoon shape. (**M**) Cocoon quality. Gray purple: gray purple, dark: dark, gray: gray, white: multiple colors; green: elliptic, light green: ellipsoid; cyan: smooth, light cyan: irregular; brown: China, light brown: Uzbekistan; blue shade degree: hatching uniformity; red shade degree: molting and exuviating uniformity; orange: male faster, light orange: together; black: black, white: multiple colors; yellow: marking, light yellow: plain; dark pink: elliptic, pink: peanut, light pink: cylindrical. Asterisks indicate significant differences with a two-tailed *t*-test: *** *p* < 0.001; n. s. *p* > 0.05.

In terms of egg color, 75% of the eggs produced by a single moth in China were pure (gray-purple) and 25% were multiple colors (gray, light gray, gray purple, gray green, light green). In contrast, eggs from Uzbekistan were composed of 67% pure color (20% gray purple, 40% dark gray, and 7% gray) and 33% multiple colors (dark gray, light gray, and light brown) (Figure 1A). Regarding egg shape, China strains had predominantly elliptic eggs, whereas Uzbekistan strains produced mostly elliptic eggs (93%) with a small proportion of ellipsoidal eggs (7%) (Figure 1B). In terms of egg attachment, 87% of Uzbekistan’s eggs were smooth, whereas the remainder (13%) were irregular; in contrast, all eggs from China were smooth (Figure 1C).

There was a significant difference in the number of eggs laid by a single moth, with an average of 505 eggs in China and 631 eggs in Uzbekistan (Figure 1D). However, there were no significant differences in the normal egg rates between the two regions (98% in China and 96% in Uzbekistan (Figure 1E). The incubation times were also similar, with an average of 10.4 days in China and 10.2 days in Uzbekistan (Figure 1F). However, the durations of the larval and pupal stages were significantly different: the larval stage was shorter in China (21.4 days) than that in Uzbekistan (28 days), and the pupal stage lasted 13.1 days in China, whereas it extended to 16.7 days in Uzbekistan (Figure 1F). China’s egg hatching was considered uniform, whereas Uzbekistan showed 67% uniform and 33% non-uniform hatching (Figure 1G). Similarly, molting and exuviating was uniform in China, whereas Uzbekistan displayed 14% uniform, 53% more uniform, and 33% non-uniform patterns (Figure 1H). Notably, all male moths from China emerged before female moths, whereas Uzbekistan had 33% of males emerging first and 67% of male and female moths emerging simultaneously (Figure 1I).

Regarding eye color, all moths from China had black eyes, whereas Uzbekistan’s moths were predominantly black (87%), with 13% exhibiting multiple colors (dark for females and white for males) (Figure 1J). Additionally, China displayed both marked (54%) and plain wings (46%), whereas Uzbekistan had solely plain wings (Figure 1K).

Both regions produced three types of cocoon shapes: elliptic, peanut, and cylindrical, albeit with different proportions: China accounted for 69% elliptic, 19% peanut, and 12% cylindrical, compared with 87%, 7%, and 6%, respectively (Figure 1L). There were no significant differences in cocoon weight and relaxation rate, with China an average of 1.52 g and 76.6%, respectively, compared to Uzbekistan’s 1.56 g and 75.4%. However, significant differences were noted in the cocoon filament length and denier count: China produced filaments averaging 560 mm and 2.33 dtex, while Uzbekistan’s filaments were longer at 994 mm and thicker at 3.21 dtex (Figure 1M).

Overall, there are numerous differences in morphological characteristics and production performance between the silkworm strains from China and Uzbekistan, which may be attributed to the different geographical environment in each region.

### 3.2. Phylogenetic Identification

**Figure 2:**
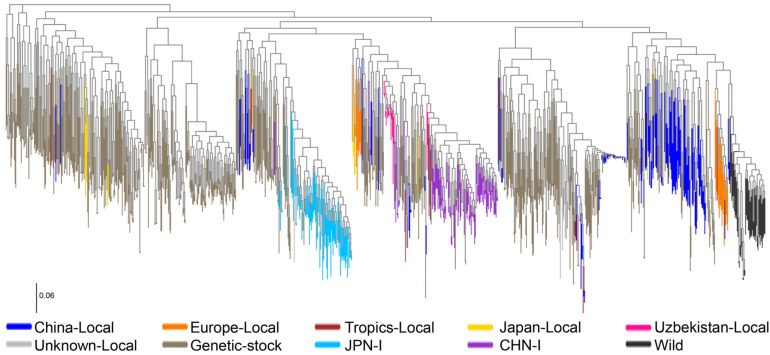
**Phylogenetic tree position of 15 representative strains from Uzbekistan and China.** China-Local (blue), local strains in China; Europe-Local (orange), local strains in Europe; Tropics-Local (brown), local strains in the tropic region (Guangdong and Guangxi provinces of China, South Asia, and Southeast Asia); Japan-Local (yellow), local strains in Japan; Uzbekistan-Local (pink), local strains in Uzbekistan; Unknown-Local (light grey), local strains without geographic information; Genetic-stock (grey), genetically selected strains; JPN-I (light blue), improved strains in Japan; CHN-I (purple), improved strains in China; Wild (black), wild silkworm.

Genome resequencing of representative local silkworm strains in Uzbekistan and subsequent comparison with 1078 silkworm genomes in open databases revealed that Uzbekistan silkworm germplasm resources are situated between local and improved strains in China (Figure 2). The China local strains, the China improved strains, and the Uzbekistan local strains were on the same root of the phylogenetic tree, indicating that they came from the same ancestor. The Uzbekistan local strains were an outgroup of the China local strains, and the China improved strains were an outgroup of Uzbekistan local strains.

### 3.3. Strain Genetic Diversity and Differentiation

**Figure 3:**
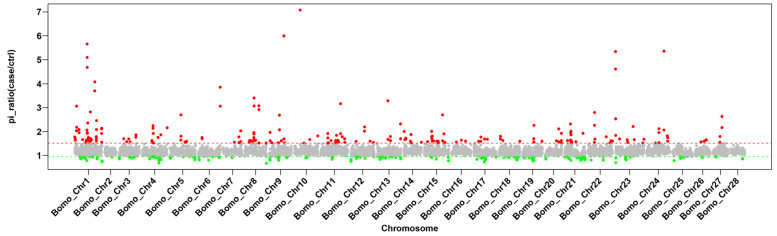
**Population nucleotide diversity of 15 representative strains from Uzbekistan and China.** Horizontal axis, chromosomes. Vertical axis, *Pi* values. *Pi* ratio = *Pi* Uzbekistan strains/*Pi* China strains. Red, the selected regions of the Uzbekistan silkworm genomes; Green, selected regions of China silkworm genomes.

**Figure 4:**
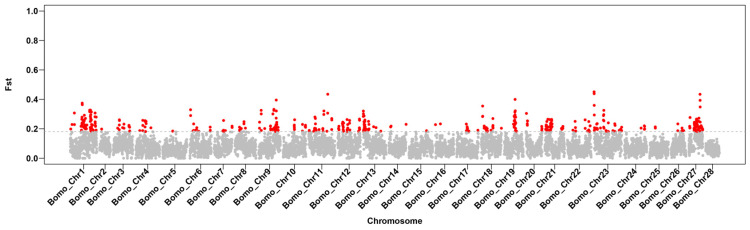
**Population differentiation of 15 representative strains from Uzbekistan and China.** Horizontal axis, chromosomes. Vertical axis, *Fst* values. Red, *Fst* values are significantly higher than the 95% confidence interval, indicating clear differentiation between two populations.

There were significant differences in production traits among 15 representative local silkworm strains in Uzbekistan. In contrast, the 15 representative China local silkworm strains were bred to suit the local climate conditions, showing smaller variations in traits. Overall, the *Pi* value for the 15 representative local silkworm strains from China was smaller than those of Uzbekistan, and the *Pi* ratio of Uzbekistan (red) was significantly more dispersed than China, especially in terms of genetic diversity on chromosome 1 (sex chromosome) (Figure 3). Similarly, we also found significant population differentiation between China and Uzbekistan by *Fst* value, and the degree of differentiation was highly concentrated in sex chromosome (Figure 4). This may be due to the fact that China silkworm have been selected to achieve morphology and production traits such as egg attachment, egg hatching, molting, and exuviating. However, the most of Uzbekistan representative local silkworm strains have not been artificially selected. *Fst* values were significantly higher than the 95% confidence interval, indicating clear differentiation between two populations. The genes in the interval were considered to be differentiation genes. GO gene function cluster analysis of differentiation genes revealed that the top 30 enriched functions mainly involve behavior regulation pathways, including reproductive behavior, egg-laying behavior, and primary sex determination (Table 1). 

## 4. Discussion

In silkworm production, morphology (egg color, egg shape, moth eye, moth wing, cocoon shape, etc.) and production performance (egg attachment, egg number, development stage, hatching uniformity, cocoon silk quality, etc.) are important indicators for evaluating germplasm resources. In China, silkworm is used not only in silk quilt, but also in silk clothing and even in medical materials. However, the predominant use remains silk quilts. in Uzbekistan. Therefore, after long-term breeding selection, the morphological diversity of silkworm strains in China is less than that in Uzbekistan, but the production traits are more suitable for silk utilization. Phylogenetic analysis reveals a close relationship between the silkworm germplasm resources of China and Uzbekistan, suggesting that silkworms in Uzbekistan may spread from China. The China local strains, China improved strains, and Uzbekistan local strains were on the same root of the phylogenetic tree. The Uzbekistan local strains were an outgroup of the China local strains. The China improved strains were an outgroup of Uzbekistan local strains. This aligns with the dissemination path of silkworm germplasm resources along the Silk Road, suggesting that Uzbekistan’s silkworm likely spread from China through the ancient Silk Road. Located around 41° North latitude, Uzbekistan presents a stark contrast to China’s primary sericulture regions, which are situated below 35° North latitude. This geographical disparity leads to substantial differences in sericulture production environments and climates, resulting in the evolution of silkworm germplasm resources with distinct productive traits over the long history of sericulture [4]. Uzbekistan’s representative local silkworm strains exhibit high egg production per moth, likely an adaptation to severe weather conditions, ensuring successful reproduction. In contrast, the representative local silkworm strains from China are renowned for their exceptional uniformity and silk quality, which can be attributed to the favorable climate in China’s main sericulture regions and a long history of artificial selection.

Genome resequencing enables a detailed identification and evaluation of germplasm resources at the molecular level [24,25]. The genetic diversity and strain differentiation exhibited by 15 representative Uzbekistan local silkworm strains are greater than those of the 15 representative China strains. This disparity may be due to China being the birthplace of silkworms, where extensive selection and breeding over many years have resulted in the development of increasingly high-quality and high-yield traits. In contrast, Uzbekistan’s local silkworm strains have a relatively shorter breeding history, and their higher variation is likely a result of their more recent natural origin, combined with significant climate differences. Behavioral regulation has emerged as a prominent distinction between the two regions, as evidenced by our Gene Ontology (GO) functional clustering analysis, which highlights differences in reproductive and egg-laying behavior. This underscores China’s emphasis on producing higher-quality cocoon silk, including silkworms that achieve a silk grade of 6A (the highest classification for raw silk). In contrast, Uzbekistan’s sericulture primarily aims to enhance production volume, with less focus on improving silk quality traits, resulting in relatively primitive and diverse characteristics.

The study showed that the nucleotide diversity and differentiation of strains tended to be consistent with the uniformity and intensity of artificial selection, such as the traditional emphasis on high quality and high yield. In addition to using conventional breeding methods to produce silkworms with superior quality and yield, China has also targeted improvements in seed production efficiency through parthenogenesis and the utilization of balanced lethal silkworms with desirable silk attributes [18,26]. Consistent with our hypothesis, the Uzbekistan strains exhibit more variation in both phenotypic traits and genetics, which is consistent with the shorter history of the sericulture and reflects the larger climatic variation from where the strains were collected.

Morphological differences, genetic diversity, and differentiation are closely interrelated. There are significant trait differences in local germplasm resources between China and Uzbekistan, particularly in the enrichment of differentiation genes on sex chromosomes. Although the study has analyzed diversity and differentiation at the genome level, it has not delved into specific target genes. In the future, the corresponding relationship between gene and morphological difference can be further analyzed. With the development of modern technologies such as gene editing, more and more traits can be achieved by modifying genes [27,28]. By further examining the genetic aspects of silkworm traits and integrating modern technologies, we aim to promote mutual advancement in the sericulture industry. Similarly, the germplasm resources of special traits were introduced and the dominant traits were integrated by hybridization. It is expected to significantly improve the quality of silk and the comprehensive benefit of sericulture.

## 5. Conclusions

Genomic resequencing for evaluation enables a deeper understanding of genetic diversity and population differentiation at the genetic level. We combined genome resequencing with an examination of production traits in local silkworm strains from China and Uzbekistan. Phylogenetic analysis of the genomic comparisons revealed that the representative local silkworm strains from Uzbekistan sit between the local and improved strains from China. Further analysis highlights distinct advantages in the silkworm strains from both countries, particularly emphasizing significant differences in their reproductive behaviors. These findings offer valuable insights for silkworm breeding initiatives aimed at enhancing cocoon quality and improving overall sericulture productivity.

## Figures and Tables

**Table 1:** The top 30 enriched GO terms in biological process.

GO ID	Description	Gene Ratio	*p* Value	*Q* Value
0072488	ammonium transmembrane transport	14/487	1.91 × 10^−9^	8.97 × 10^−6^
0050795	regulation of behavior	38/487	9.28 × 10^−9^	2.18 × 10^−5^
0015697	quaternary ammonium group transport	14/487	3.02 × 10^−8^	4.74 × 10^−5^
0015695	organic cation transport	16/487	2.28 × 10^−7^	2.68 × 10^−4^
0015870	acetylcholine transport	11/487	6.61 × 10^−7^	5.18 × 10^−4^
1901374	acetate ester transport	11/487	6.61 × 10^−7^	5.18 × 10^−4^
0098656	monoatomic anion transmembrane transport	35/487	1.33 × 10^−6^	8.96 × 10^−4^
0015871	choline transport	8/487	3.95 × 10^−6^	2.32 × 10^−3^
0090328	regulation of olfactory learning	9/487	4.54 × 10^−6^	2.37 × 10^−3^
0032223	negative regulation of synaptic transmission, cholinergic	8/487	1.44 × 10^−5^	6.77 × 10^−3^
0006820	monoatomic anion transport	44/487	2.41 × 10^−5^	1.03 × 10^−2^
0019098	reproductive behavior	35/487	3.93 × 10^−5^	1.54 × 10^−2^
0015711	organic anion transport	37/487	4.45 × 10^−5^	1.61 × 10^−2^
0060148	positive regulation of post-transcriptional gene silencing	8/487	7.80 × 10^−5^	2.44 × 10^−2^
1900370	positive regulation of post-transcriptional gene silencing by RNA	8/487	7.80 × 10^−5^	2.44 × 10^−2^
0034383	low-density lipoprotein particle clearance	7/487	9.59 × 10^−5^	2.82 × 10^−2^
0060147	regulation of post-transcriptional gene silencing	11/487	1.12 × 10^−4^	2.92 × 10^−2^
1900368	regulation of post-transcriptional gene silencing by regulatory ncRNA	11/487	1.12 × 10^−4^	2.92 × 10^−2^
2000677	regulation of transcription regulatory region DNA binding	8/487	1.37 × 10^−4^	3.38 × 10^−2^
0018991	egg-laying behavior	20/487	1.55 × 10^−4^	3.65 × 10^−2^
0007613	memory	27/487	1.82 × 10^−4^	3.95 × 10^−2^
0060359	response to ammonium ion	17/487	1.85 × 10^4^	3.95 × 10^−2^
0032222	regulation of synaptic transmission, cholinergic	10/487	2.17 × 10^−4^	4.44 × 10^−2^
0007538	primary sex determination	7/487	2.47 × 10^−4^	4.83 × 10^−2^
0044057	regulation of system process	36/487	3.02 × 10^−4^	5.59 × 10^−2^
0050650	chondroitin sulfate proteoglycan biosynthetic process	6/487	3.09 × 10^−4^	5.59 × 10^−2^
0048521	negative regulation of behavior	8/487	4.51 × 10^−4^	7.85 × 10^−2^
0007542	primary sex determination, germ-line	5/487	4.74 × 10^−4^	7.96 × 10^−2^
0060966	regulation of gene silencing by regulatory ncRNA	11/487	6.52 × 10^−4^	1.06 × 10^−1^
1905606	regulation of presynapse assembly	5/487	7.04 × 10^−4^	1.06 × 10^−1^

## Data Availability

Data are contained within the article.
